# Two Efficient Techniques to Find Approximate Overlaps between Sequences

**DOI:** 10.1155/2017/2731385

**Published:** 2017-02-15

**Authors:** Maan Haj Rachid

**Affiliations:** Qatar University, P.O. Box 2713, Doha, Qatar

## Abstract

The next-generation sequencing (NGS) technology outputs a huge number of sequences (reads) that require further processing. After applying prefiltering techniques in order to eliminate redundancy and to correct erroneous reads, an overlap-based assembler typically finds the longest exact suffix-prefix match between each ordered pair of the input reads. However, another trend has been evolving for the purpose of solving an approximate version of the overlap problem. The main benefit of this direction is the ability to skip time-consuming error-detecting techniques which are applied in the prefiltering stage. In this work, we present and compare two techniques to solve the approximate overlap problem. The first adapts a compact prefix tree to efficiently solve the approximate all-pairs suffix-prefix problem, while the other utilizes a well-known principle, namely, the pigeonhole principle, to identify a potential overlap match in order to ultimately solve the same problem. Our results show that our solution using the pigeonhole principle has better space and time consumption over an FM-based solution, while our solution based on prefix tree has the best space consumption between all three solutions. The number of mismatches (hamming distance) is used to define the approximate matching between strings in our work.

## 1. Introduction

The next-generation sequencing (NGS) technology creates a new type of challenges. The output of NGS is a huge number of sequences (reads) which require further processing. The generated sequences represent segments from multiple copies of the original genome. An overlap-based assembler, such as SGA [1] and Readjoiner [2], finds overlaps between these reads in order to build a string graph which will be the input for the assembly stage. The problem of finding overlaps between each ordered pair of reads is commonly called the all-pairs suffix-prefix problem (APSP).

For a given group of strings *G* = *S*_1_, *S*_2_,…, *S*_*k*_, solving APSP is to find the largest (longest) suffix-prefix match for each ordered pair in *G*. Gusfield et al. presented a solution for APSP in an optimal time using a generalized suffix tree [3]. For a text *T*, a suffix tree ST is a tree in which every suffix in *T* is represented by a path from the root to a leaf. The drawback for this solution is the high consumption of memory since the best implementation for suffix tree consumes 20*n* bytes for a text of size *n* characters [4]. Ohlebusch and Gog [5] presented a solution for APSP using an enhanced generalized suffix array with an optimal time but with much less space than the one utilizing a suffix tree. The algorithm was practically improved [6].

A suffix array SA of a text *T* is an array containing values which range from 1 to *n* and represent the text positions of the lexicographically sorted suffixes of *T*. An LCP array is an array storing the length of the largest common prefix between every two consecutive lexicographically sorted suffixes in *T*. An enhanced suffix array is a suffix array and an LCP array. In both solutions (suffix tree and enhanced suffix array), reads are first concatenated in one string *S*. In *S*, every two consecutive reads are separated by a distinct character which is not repeated anywhere in *S*; then the data structure is built for the resulting text. The word “generalized” indicates that the data structure is built from all reads.

Compressed data structures have also been utilized to solve APSP. Simpson and Durbin used FM index [7] to solve APSP. Sadakane suffix tree [8] and run-length compressed suffix array (RLCSA) are also utilized to solve APSP ([9] and [10], resp.). A very recent work [11] showed that a compact prefix tree can be used to solve APSP efficiently in terms of time and space.

One of the most important fields for applying APSP is genome assembly. Assemblers can be classified depending on the type of graph they are building as follows:

- They either build a string graph in which a node represents a read and an edge represents an overlap between two reads. Such assembler is called an overlap-based assembler. Since finding the original genome using a string graph by finding a path that visits every node exactly once is an NP-problem, such assemblers use techniques to solve a reduced version of this problem in the assembly stage [12].
- Or they build a de Bruijn graph in which nodes are b-1 mers of the reads and the edges correspond to the overlaps of size b-2 between two b-1 mers, where b is a fixed value less than the length of a read. The assembler then finds the original genome using the de Bruijn graph by finding a path which visits every edge in the graph only once. However, this graph may have many alternative paths, and therefore it is the first step in creating a good draft assembly [13].

A traditional overlap-based assembler would first filter the set of reads by removing redundant reads and applying approximate string-matching methods to detect and to correct errors in these reads. Then it would find exact overlaps between the prefiltered reads by solving APSP. However, a few researches tackled the approximate version of APSP (AAPSP) such as the work of [14]. The advantage of this trend is to avoid the error-correction preprocessing steps which use time-consuming approximate string-matching techniques. Valimaki et al. utilized a compressed data structure (FM index) with the backward backtracking technique to solve AAPSP. It also takes advantage of suffix filters which were introduced by [15] and improved by [16].

## 2. Objectives

In this work, we present two techniques to solve AAPSP. The first utilizes a compact prefix tree in solving AAPSP, while our second technique takes advantage of the well-known pigeonhole principle and the minimal length for an overlap in order to identify potential overlap matches. We compare our work with the work presented by [14].

We first explain our methods in Section 3.  Section 4 demonstrates our experiments and discusses our results. We draw our conclusion in Section 5.

## 3. Methods

### 3.1. Definitions

#### 3.1.1. Compact Prefix Tree

**Figure 1:**
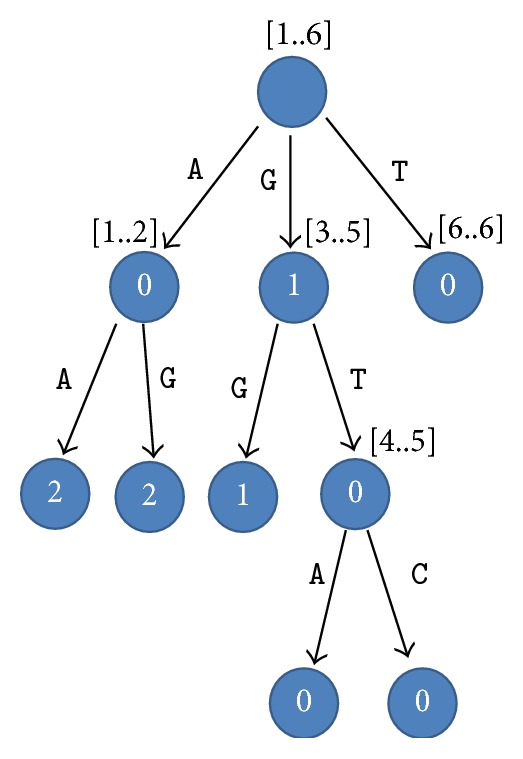
The compact prefix tree for strings *S*_1_ = AGGT, *S*_2_ = GGTC, *S*_3_ = AATG, *S*_4_ = GGTA, *S*_5_ = TTAC, and *S*_6_ = GGGC. The range above each node represents the reads which share the prefix up to this node. The value inside a node indicates the *chain*_*len* value of this node. For example, the range [4..5] indicates that the reads 4 and 5 share the prefix GGT. The prefix GGT can be obtained by concatenating all the labels of the edges starting from the root and ending with the node ([4..5]). Note that the second G in GGT is obtained from the text since *chain*_*len* = 1. The numbers inside the ranges are the new identifiers of the strings after sorting, not the original identifiers.

The words “read” and “sequence” are used interchangeably in this work. A read is a string of characters over an alphabet Σ = {A, C, G, T}. We define a prefix tree *P* for a group of reads *G* as a tree in which every read in *G* is represented by a path from the root to a leaf. Every edge in *P* is labeled with one of the four characters: A, C, G, or T. Every node *v* has an interval [*r*_*i*_..*r*_*j*_] where *r*_*i*_..*r*_*j*_ are the identification numbers of the reads which share the same prefix up to *v* (assuming that the reads are sorted). Since an edge is labeled by one character and reads in one range [*r*_1_..*r*_*j*_] may share a substring *sub* with a length *subl* > 1, every node has also a value *chain*_*len* = *subl* − 1, which represents the length of a common substring between all reads in the range [*r*_*i*_..*r*_*j*_]. The benefit of this value is to avoid building unnecessary nodes for substrings which are shared by reads in one range.  Figure 1 shows an example for a compact prefix tree.

The compact prefix tree can be built in *O*(*n*) time where *n* is the total length of all reads. The space consumption for building this tree is *O*(*k*) where *k* is the count of the reads. Clearly, the presence of the input reads is required since this data structure is not a self-index data structure.

### 3.2. Approximate Matching

An approximate matching between two strings can be expressed by the edit distance. The edit distance between strings *T*_1_ and *T*_2_ is defined as the minimum number of insertions, deletions, and replacements of symbols to transform string *T*_1_ into *T*_2_ [17]. Hamming distance is another way to describe an approximate match. The hamming distance between strings *T*_1_ and *T*_2_ is the number of mismatching symbols between strings *T*_1_ and *T*_2_. A string *T*1 is considered an approximate match to *T*2 if the edit distance (or the hamming distance) between the two strings is ≤ *z*, where *z* is the number of allowed insertions, deletions, and replacements (or mismatches when hamming distance is used) to transform *T*_1_ to *T*_2_.

#### 3.2.1. Pigeonhole Principle

The basic idea behind the pigeonhole principle is that if there is an approximate matching between two strings *S*_1_, *S*_2_, then there must be an exact matching between them with a smaller size. The size of the available exact matching between the two strings is determined by the hamming distance (or edit distance) between them. Given that *S*_1_ differs from *S*_2_ by *m* characters, if we divide *S*_1_ into *m* + 1 parts, then one of these parts will exactly match a part in *S*_2_. The principle can easily be proved by contradiction.

The crucial benefit of the pigeonhole principle is the identification of candidates for an approximate matching between the two strings. As a result, extremely fast exact matching algorithms and techniques are used to find these candidates and the time-consuming dynamic programming technique is only used to verify if a candidate is a part of an approximate matching between the two aligned strings.

The principle is also known as seed-and-extend. It is the base of many genome analysis algorithms such as Basic Local Alignment Search Tool (BLAST) and many works such as [18] utilized this concept in sequence alignment. In this work, we employ this principle to find overlaps between reads.

#### 3.2.2. AAPSP

It is easy to define APSP because of its exact nature; however, defining AAPSP may not be very clear since the preference may differ between the length of the match and the number of mismatches; that is, a suffix-prefix match with a length of 20 and 7 mismatches may be better than one with a length of 10 and 4 mismatches. Reference [19] demonstrated different interests in approximate matching for a bioinformatician. In this study, we adopt the following interest. For each ordered pair of the input reads, we target the largest suffix-prefix match with a maximum of *m* allowed mismatches. Nevertheless, we show that our solution can be easily modified to cover most other definitions.

### 3.3. Solving AAPSP Using a Compact Prefix Tree

The work of [11] describes the technique to solve APSP using a compact prefix tree. Every suffix in every read is matched with a path in the prefix tree (if there is one). The algorithm presented in the work of [11] takes advantage of the minimal length for a suffix-prefix match min by ignoring all suffixes which are shorter than *min*. The time consumption for solving APSP is *O*(*kℓ*^2^) where *k* is the number of reads and *ℓ* is the maximum size of a read. While *k* may vary in practice from hundreds of thousands to hundreds of millions, *ℓ* is usually less than a thousand.

Our first technique employs a compact prefix tree to solve AAPSP. When *m* mismatches are allowed, every suffix *S* in every read is aligned with every path *p* in the tree, where *p* is a path from the root to a leaf. In the attempt to match a suffix *S* with a path *p* in the tree, mismatches are counted up to a threshold. If a threshold is reached before reaching the end of *S*, then *S* does not represent a match between a suffix and a prefix. The pseudocode is shown in Algorithm 1. The following variables are used in Algorithm 2.

- *mislimit* is the number of allowed mismatches.
- *mismatch* is the so-far number of mismatches.
- *v* is the current character in *Su* (current suffix).
- *current*_*char* is the current character to compare with in the prefix tree. It can be found by calculating the length of the path from the root to the current node. Accordingly, the current read mentioned in the algorithm is one of the reads which are included in the range of current node.
- *current*_*node* is a pointer to the current node in the prefix tree.
- *local*_*position* is used to check if the comparison is done inside a node by comparing *local*_*position* with the *chain*_*len* of the *current*_*node*.
- *current*_*node*.*child*(*c*) returns true if the current node has a child with a character *c*, that is, there is an edge coming from current node towards another node and labeled by a *c*.

The code is simple. For every suffix *Su* in every read, the *findAllPairs* procedure is called. If the end of *Su* is reached, then *Su* represents a suffix-prefix match between the read that contains it and every read which is included in the interval of the current node in the prefix tree (lines (3)–(6)). If this is not the case, then we distinguish two cases:

- The comparison is done between a character in *Su* and a character indicated by the value of *chain*_*len* in the current node in the tree. It happens when the *chain*_*len*≥*local*_*position* (lines (8)–(17)).
- A character in *Su* is compared with a label of an edge. In this case, *local*_*position* exceeds the value of *chain*_*len* of the current node. In this case, we test every child of the current node using a recursive call unless the allowed mismatches are exhausted (lines (18)–(23)).

Let us try to match suffix GGC from string 6 with a path in Figure 1 assuming that the number of allowed mismatches is 1. The first path starts with AA, so there is no match. The second path starts with AGG, so we can see that the comparison will fail when reaching the third character since we will have 2 mismatches. The third path starts with GGG which is a match since it differs from GGC by only one character. Accordingly, GGC is a suffix-prefix match, but it is not relevant since it involves the same read. The fourth path is GGT which is a match since it differs from GGC by only one character. Accordingly, GGC represents a suffix-prefix match between string 6 and each of string 2 and string 4 (which are identified as 4 and 5 after sorting). Finally, the last path does not present any match.

The difference between the two usages of compact prefix tree in solving APSP and AAPSP is clear. In APSP, every suffix is matched with a path in the tree, while in AAPSP, every suffix is tested against every path in the tree.

Given that there are *ℓ* suffixes in a read, all suffixes of a read can be processed using a compact prefix tree in *O*(*ℓ*^2^*k*) where *k* is the number of reads. The time complexity for the solution is *O*(*n*^2^) in the worst case where *n* is the total size of all reads. However, in practice, the solution runs much faster than the worst case. The space complexity is bounded by the size of the text which is *O*(*n*log⁡Σ), since the construction of the prefix tree requires only *O*(*k*) space [11].

#### 3.3.1. Solving AAPSP Using Pigeonhole Principle

Our second technique takes advantage of the pigeonhole principle and the minimal length for an overlap in order to identify the candidate suffixes which can be approximate suffix-prefix matches between pairs of reads. Let *min* be a minimal length for an overlap (i.e., a suffix-prefix match with a length *min* will not be considered). If a suffix *S* is an approximate suffix-prefix match with a threshold *m*, then its prefix of length *min* has to have a hamming distance ≤*m* when aligned with a prefix with the same length of some read *r*. Accordingly, if *S* is divided into *m* + 1 parts, then one of these parts exactly matches a corresponding part of a prefix *p* of a read *r*. We then compare all remaining parts of *S* with their corresponding parts in *p*.

Accordingly, the technique can be summarized as follows:

- Divide the prefix of length min for each read into *m* + 1 parts.
- Add each part *p* from the prefix of read *r* to an index which has entries of type 〈*key*, *L*〉 where *L* is a list of reads. Accordingly, if *p* is already in the index, *r* will be added to an existed entry (in its *L* list); otherwise, a new entry 〈*p*, {*r*}〉 will be added to the index.
- Every suffix *S* that can be an acceptable overlap match will be tested. *S* is divided into *m* + 1 parts and every part is searched for in the index. If a part *p* is found at position *i* in the index, we investigate every read *r*_1_ in *L*_*i*_. We compare all characters that precede *p* in *S* with their corresponding characters in *r*_1_. If the threshold is not reached, we compare all characters after *p* in *S* with their corresponding characters in *r*_1_ until the threshold of mismatches is reached. If the end of *S* is reached without exceeding the limit of mismatches, then *S* is reported as an approximate overlap between *r* (the read which contains *S*) and *r*_1_.

**Figure 2:**
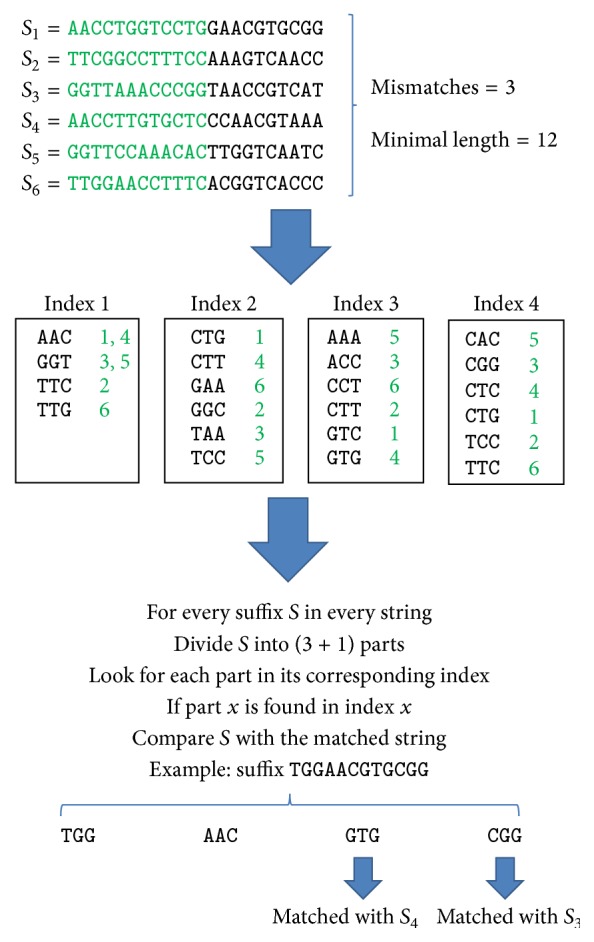
A demonstration for the pigeonhole principle. Every prefix of size *min* from every read is divided into 4 parts. Each part is inserted in its appropriate index. Then, each candidate suffix *S* is divided into 4 parts and each part is searched for in the corresponding index. If there is a match, then we compare *S* with the prefix of the matching string.

Figure 2 demonstrates the basic concept. The pseudocode is shown in Algorithm 3. The worst case time complexity is *O*(*kℓ*^2^), but, in practice, the solution runs much faster. The index can have (*m* + 1)*k* entries. All entries may have up to (*m* + 1)*k* values (in all *L* lists). Therefore, the space complexity is bounded by the size of the text which is *O*(*n*log⁡Σ) where *n* is the total length of all reads and Σ is the size of the alphabet.

One drawback for this algorithm is the repetition in reporting the overlaps. The same overlap may be reported more than once since each matching part may end up reporting an overlap. We used a hash table to keep track for the overlaps which are reported. The hash table is cleared for every candidate suffix *S*.

#### 3.3.2. Implementation Notes

We used an unordered map 〈*key*, *L*〉 to build our index, where *key* is a part of a prefix of size min in a read and *L* is a list of reads. To make the implementation simple, we used an index for each part. Accordingly, if *m* = 3, we use 4 indices and each part *p* in every prefix of size min in every read is inserted into its appropriate index (i.e., part 1 into index 1). The prefix of length *min* in each suffix *S* is divided into *m* + 1 parts and each part *p* is searched for in its appropriate index. If a match is found, the list of reads which is associated with the key (*p*) is retrieved and we start comparing whatever before and after part *p* in *S*.

#### 3.3.3. Supporting Other Flavors of Approximate Matching

In this study, we use the hamming distance concept to define an approximate overlap match. However, our solutions support other matching types such as spaced seeds, subset seeds, and edit distance [19]. Spaced seeds can be described as a mask M which is represented by a string over the alphabet {0,1} where 0 indicates an allowed mismatch position. For example, string ACGCTATTG with a mask 011 accepts GTG, CTG, and GTACTG as suffix matches (we apply the mask cyclically in GTACTG twice since GTACTG is longer than the mask).

A slight modification in Algorithm 1 is sufficient to fulfill the spaced seed type. A variable to track the current element of the mask would be needed. The condition in lines (9)–(11) can be easily extended in order to validate the comparison and a return statement should be executed if a mismatching occurs and the current element of the mask is 1. A similar modification should be done to the condition in line (24); no recursive call is executed if the current element of the mask is 1. In Algorithm 3, similar modifications are required for comparisons in lines (7) and (9).

With subset seeds, we specify the types of mismatches that are allowed at each position. For instance, {{*a*, *t*}, {*c*, *g*}} allows a,t mismatches and *c*, *g* mismatches only. This form is also easy to incorporate in our solutions by adding more checking before considering the case as a mismatch. If the case is not included in the group of allowed mismatches, we ignore the candidate suffix and move on to the next one.

The edit distance is not supported in our solution.

## 4. Experimental Evaluation

### 4.1. Experimental Setup

We implemented two C++ solutions to solve AAPSP using our two techniques. They use openMP to support multithreading. The used parallelizing technique is based on dividing reads equally between threads. We used /usr/bin/time to measure the time and space. The source code for our solutions can be downloaded from: https://github.com/maanrachid/Codes/blob/master/AAPSP.tar.

The implementation is tested on two types of machines:

- A modest 2-core virtual machine with 1 GB RAM and less than 10 GB hard disk running on 2.00 GHZ CPU: we ran randomly generated data sets on the machine. The random data is generated by a program which creates *k* reads with a total of length *n*. *n* and *k* are inputs from the user. Testing on such machine is to demonstrate our solutions' ability to find overlaps even with limited resources. The minimal length of an approximate overlap in our experiments is 30.
- An 8-core AWS node for testing large real samples: testing our solutions on this node evaluates their ability to handle large data set. Eight threads are used in all experiments. The minimal length of an approximate overlap in our experiments is 30.

Table 1 describes data sets used by the experiments. We obtained our real data from PubMed (http://www.ncbi.nlm.nih.gov/pubmed) and Citrus Genome databases (http://www.citrusgenomedb.org).

### 4.2. Experiments Results

We compare the time and space consumption for prefix tree (PT), pigeonhole (PH), and FM [14] solutions when used to solve AAPSP on a modest machine with randomly generated data. Tables 2 and 3 show the result of our testing. The number of threads which are used in our experiments is 2, the minimal length of an overlap is 30, and the output option is set on (produce output).

It is very clear that PH has the best results in terms of time. PT has better results than FM in the first two data sets and worse performance with the last one. This is due to the fact that the read in the last data set has a length of 500 (while the length of a read in the first two data sets is 100). PT favors short reads since the minimal length of an overlap can be utilized better (more comparisons can be skipped when the reads are short). In terms of space, PT has the best consumption with a clear advantage for PH over FM.

We test our solutions with real and large data sets on an AWS node with high capabilities (8 cores, 60 GB RAM, and 200 GB hard disk). The required time and space are shown in Tables 4 and 5. The minimal length for an overlap is 30, the number of threads is 8, and the output option is set on.

Clearly, pigeonhole solution demonstrates superior results in terms of time; however, prefix tree consumes less space in all data sets. Despite its low-space requirement, the brute force nature of the prefix tree solution causes a high time consumption.

We should mention that FM performs some additional tasks such as handling the N character or finding the overlaps for the reverse complement. That may affect the time and space consumption. However, it will not doubt the advantage of PH over FM in terms of time and space and the advantage of PT over FM in terms of space since the differences are too big to be interpreted by such factors.

## 5. Conclusion

Both our solutions can be used efficiently to solve AAPSP with a relatively small number of mismatches. It has been shown that the pigeonhole solution is superior in terms of time and has better space consumption than FM, while the prefix tree achieves the best space consumption between all three solutions. Both our solutions can efficiently contain other flavors of approximate matching with the exception of edit distance (deletion and insertion).

It would be great if these tools can be extended to find overlaps using the edit distance. It may also be interesting to find out how efficient our tools are when integrated with other components in an overlap assembler.

## Figures and Tables

**Algorithm 1:**
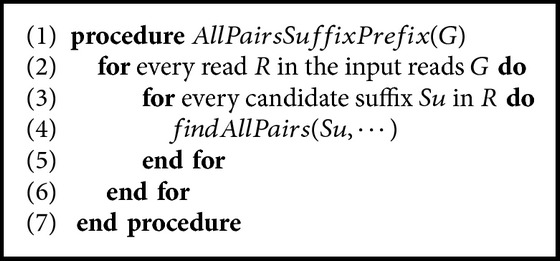
Solving AAPSP using a prefix tree.

**Algorithm 2:**
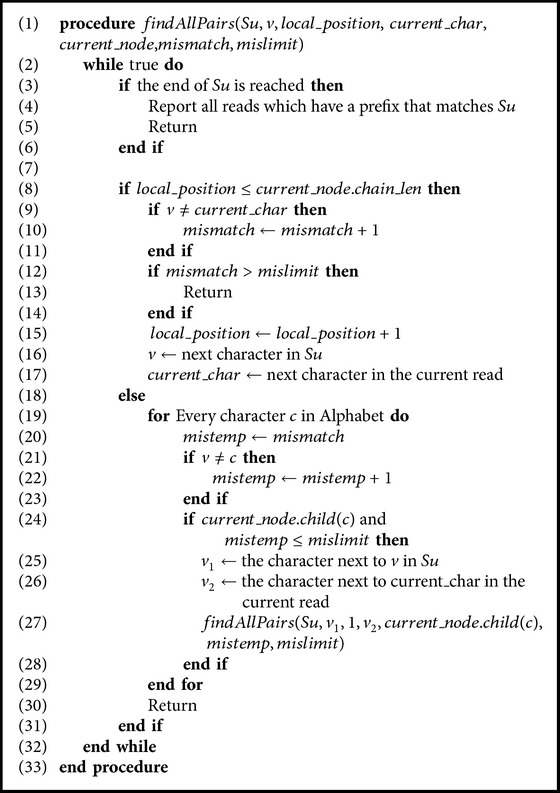
Solving AAPSP using a prefix tree.

**Algorithm 3:**
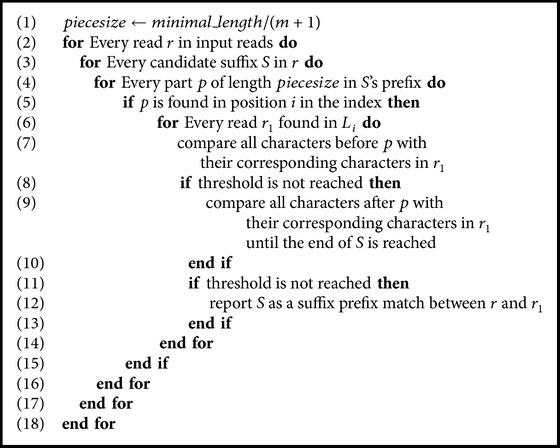
Solving AAPSP using pigeonhole principle.

**Table 1:** Data sets used in experiments.

Data Set	Size	# of strings
Random data	1 MB–5 MB	5000–50000
Homo sapiens exome (SRR500004)	1.1 GB	15 M
*E. coli* (SRR2244250)	302 MB	502,172
*C. elegans *	167 MB	334,465
*Citrus clementina *	104 MB	118,365
*Citrus sinensis *	154 MB	208,909
*Citrus trifoliata *	46 MB	62,344

**Table 2:** Time consumptions for prefix tree (PT), pigeonhole (PH), and FM solutions to find approximate overlaps using different values for allowed mismatches (*m*). Time is shown in seconds.

Data set	*m* = 1	*m* = 2	*m* = 3	*m* = 1	*m* = 2	*m* = 3	*m* = 1	*m* = 2	*m* = 3
	PT	PT	PT	PH	PH	PH	FM	FM	FM
1 MB	1.8	10.6	45	0.5	0.5	1.2	3	17	120
5 MB	15	126.3	672	2.4	3.3	14	17	127	960
2.5 MB	4	27	109	1.34	2	3	4	13	82

**Table 3:** Time consumptions for prefix tree (PT), pigeonhole (PH), and FM solutions to find approximate overlaps using different values for allowed mismatches (*m*). Time is shown in seconds.

	PT	PH	FM
1 MB	2.3	4	9
5 MB	6.5	14.5	40
2.5 MB	2.5	4	21

**Table 4:** Time consumptions for prefix tree (PT), pigeonhole (PH) and FM solutions to find approximate overlaps when real data is used on a capable AWS node. Time is shown in seconds.

Data Set	*m* = 1	*m* = 2	*m* = 3	*m* = 1	*m* = 2	*m* = 3	*m* = 1	*m* = 2	*m* = 3
	PT	PT	PT	PH	PH	PH	FM	FM	FM
*Citrus clementina *	122	1094	5749	16	42	392	54	200	1377
*Citrus sinensis *	233	2229	12053	49	361	1352	100	442	3501
*Citrus trifoliata *	30	223	1069	10.5	45	158	24	92	660
*C. elegans *	381	3681	21390	104	241	1682	186	792	4806
SRR2244250	757	8111	30234	49	151	6023	357	2340	16161
SRR500004	3502	20342	90321	252	1414	8752	1787	6813	44907

**Table 5:** Space consumptions for prefix tree (PT), pigeonhole (PH), and FM solutions to find approximate overlaps when real data is used on a capable AWS node. Space is shown in MB.

	PT	PH	FM
*Citrus clementina *	65	66	807
*Citrus sinensis *	86	110	803
*Citrus trifoliata *	34	37	371
*C. elegans *	110	138	783
SRR2244250	230	298	2416
SRR500004	727	818	1013
